# Supplementary figures and images for: Microarray Gene Expression Analysis of Lesional Skin in Canine Pemphigus Foliaceus
Source: Vet Sci. 2024 Feb 14;11(2):89. doi: 10.3390/vetsci11020089 (PMC10893259; doi:10.3390/vetsci11020089)

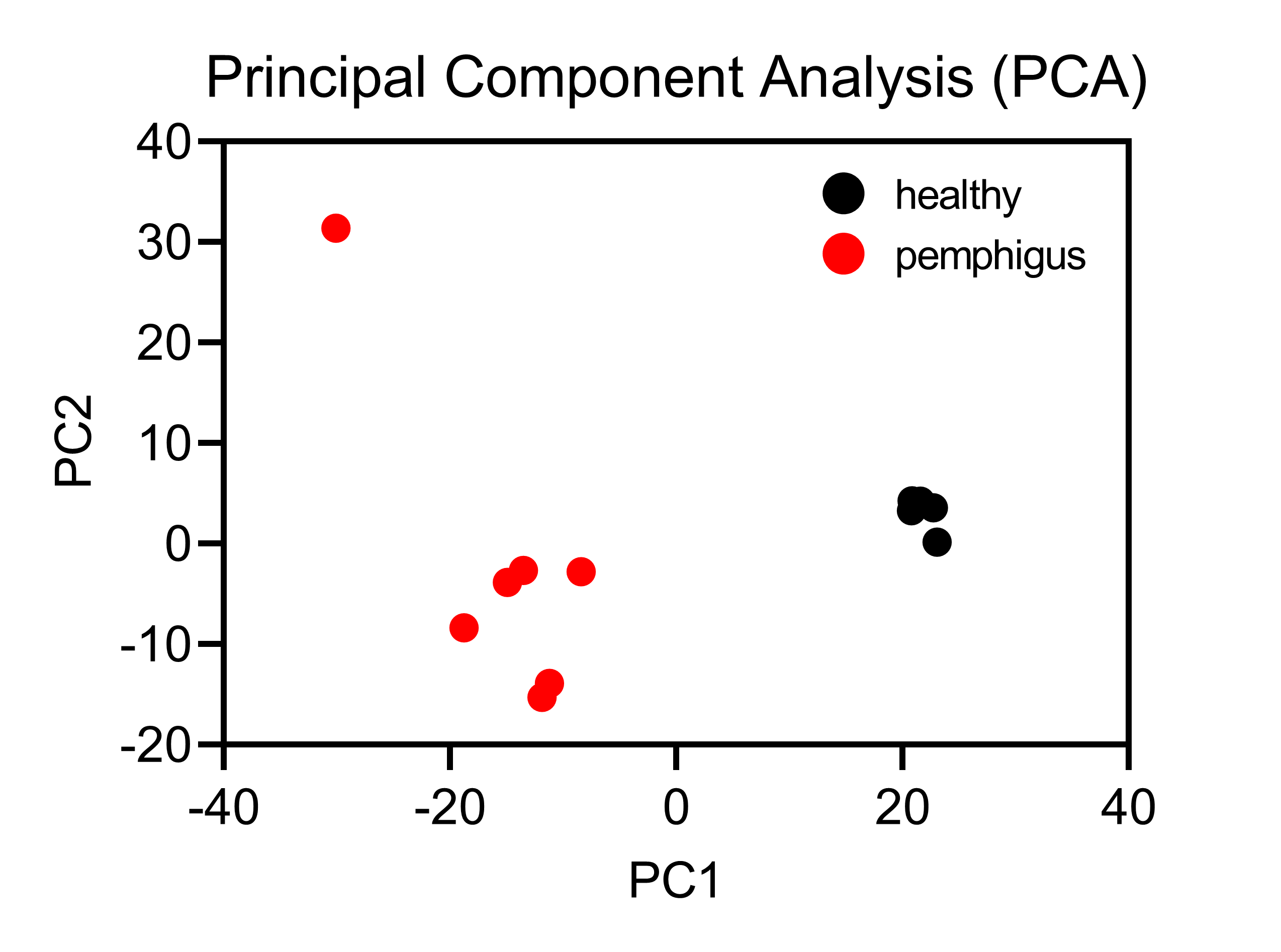

Supplement: Supplementary file 1 [file vetsci-11-00089-s001.zip › Figure_Suppl_1.PCAScoresPlot.tif]
